# Combined Toxicity of Multi-Walled Carbon Nanotubes and Cu^2+^ on the Growth of Ryegrass: Effect of Surface Modification, Dose, and Exposure Time Pattern

**DOI:** 10.3390/nano14211746

**Published:** 2024-10-30

**Authors:** Wenwen Xie, Cheng Peng, Weiping Wang, Xiaoyi Chen, Jiaqi Tan, Wei Zhang

**Affiliations:** 1 State Environmental Protection Key Laboratory of Environmental Risk Assessment and Control on Chemical Process, School of Resource and Environmental Engineering, East China University of Science and Technology, Shanghai 200237, China; y30221128@mail.ecust.edu.cn (W.X.); y30211101@mail.ecust.edu.cn (W.W.); y30231083@mail.ecust.edu.cn (X.C.); wzhang@ecust.edu.cn (W.Z.); 2 Shanghai Institute of Pollution Control and Ecological Security, Shanghai 200092, China; 3 Department of Biological Sciences, Louisiana State University, Baton Rouge, LA 70803, USA

**Keywords:** multi-walled carbon nanotubes, copper, toxicity, ryegrass, combined exposure

## Abstract

The escalating release of multi-walled carbon nanotubes (MWCNTs) into the environment has raised concerns due to their potential ecotoxicological impacts. However, their combined phytotoxicity with heavy metals such as copper (Cu) is still unclear. This study investigated the individual and combined toxic effects of MWCNTs (MWCNT, MWCNT-OH, and MWCNT-COOH) and Cu^2+^ on ryegrass (*Lolium multiflorum*), uniquely considering different addition orders. The results show that Cu severely inhibited the growth of ryegrass while MWCNTs exhibited a hormesis effect on ryegrass. When MWCNT and Cu were combined, the malondialdehyde (MDA) content in ryegrass showed a 32.39% increase at 20 mg/L MWCNT exposure, suggesting reduced oxidative stress. However, at the higher concentration of 1000 mg/L, it led to a significant 75.22% reduction in ryegrass biomass. MWCNT-COOH had the most pronounced effect, reducing the total chlorophyll content by 39.76% compared to unmodified MWCNT and by 10.67% compared to MWCNT-OH (500 mg/L). Additionally, pre-induced MWCNTs might alleviate the Cu in the plant by 23.08–35.38% through adsorption in the nutrient solution. Small molecule organic acids and amino acids primarily mediated the response to environmental stress in ryegrass. This research provides crucial insights into understanding the complex interactions of MWCNT and Cu^2+^ and their combined effects on plant ecosystems.

## 1. Introduction

Carbon nanotubes (CNTs), characterized by their one-dimensional tubular structure, hold great potential within this material family due to their high mass transport efficiency and vast specific surface area. This has led to a drastic increase in their production over the last decade [1,2]. Multi-walled carbon nanotubes (MWCNTs) are one of the most commonly used carbon nanomaterials among CNTs. Due to their high porosity and exceptional adsorption capacity, driven by electrostatic interactions with adsorbates, CNTs are highly efficient for a wide range of critical applications, such as energy capture and storage, sensing technologies, catalysis, transistors, and environmental protection [3]. CNTs are increasingly released into the environment, contaminating soil, water, and air throughout their lifecycle during production, application, and disposal. Therefore, concerns over their potential toxicity to plants, as well as their broader impacts on environmental and human health, have raised concerns about whether their associated risks outweigh the benefits.

The toxic effects of nanoparticles on plants are well known. For example, TiO_2_ nanoparticles and silver nanoparticles at the concentration of 100 mg/L have been found to inhibit the germination of lettuce (*Lactuca sativa*), cucumber (*Cucumis sativus*), and cauliflower (*Brassica oleracea*) [4]. Like metallic nanoparticles, CNTs can negatively impact not only seed germination, plant growth, and development [5] but also photosynthesis, cell structure, and even gene expression [6]. MWCNTs, in particular, have been observed to inhibit cell growth and viability, reduce chlorophyll content, and decrease enzyme activities at concentrations ranging from 10 to 600 mg/L [7].

Moreover, MWCNTs introduced into the environment undergo various forms of aging processes, which can alter their surface properties and charge, significantly influencing their physicochemical characteristics [8]. Consequently, their behavior and toxic potential in the environment can be changed [9]. At a concentration of 20 mg/L, positively charged and neutral MWCNTs exhibited inhibitory effects on maize and reduced transpiration and biomass growth in soybeans. Through a comparative analysis of the rate and extent of transpiration and biomass growth between different MWCNT types, negatively charged MWCNT-COOHs showed the weakest inhibitory effect compared to their positively charged and neutral counterparts [10]. The presence and type of CNTs significantly influenced pesticide availability to lettuce seedlings. Compared to non-functionalized CNTs, the effects of amino-functionalized CNTs were significantly less pronounced, with reduced pesticide content in both roots and shoots [11]. When plants were subjected to external stress, MDA levels tended to increase, indicating oxidative damage to the cell membranes, which led to a reduction in chlorophyll content, inhibition of photosynthesis, and ultimately a decrease in biomass [12]. Yet the phytotoxicity of MWCNTs with different functional groups remain unclear.

Additionally, it is worth noting that CNTs and copper (Cu), especially when acting as catalysts, are usually and simultaneously used in the industrial production. Cu is an essential trace element for plant growth and development but becomes particularly toxic at excessive levels, inhibiting growth and potentially causing severe plant mortality [13,14]. Excess Cu^2+^ affects cellular pigments and biochemical processes, inhibiting chlorophyll synthesis and accelerating chlorophyll degradation [15,16]. It also compromises cell membrane integrity and function, disrupting ion absorption, transport, and osmotic regulation, leading to metabolic disturbances [17]. Moreover, ROS generation and oxidative stress induced by heavy metals (HMs) can increase carbonylated proteins and MDA concentrations in injured tissues [18]. Interestingly, the toxicity of metals is also correlated to their forms, including bulk, nanoparticles, and ionic forms. Exposure to nCu, bulk Cu, nCuO, bulk CuO, Cu(OH)_2_, and CuCl_2_ reduced the root length of lettuce (*Lactuca sativa*) and the concentrations of P and Fe in the shoots [19]. Furthermore, nCuO caused more physiological impairments to 70-day-old Rosie and green bok choy (*Brassica rapa*) than bulk CuO and CuCl_2_ treatments [20].

Due to the high adsorption capacity of CNTs for HMs, CNTs can adsorb HMs in water through strong surface complexation reactions [21], altering the bioavailability and toxicity of HMs in the environment [22,23]. However, the adsorption process, in turn, is likely to influence the characteristics of the CNTs themselves [24]. MWCNTs have been evidenced to alleviate the toxicity of cadmium and arsenic in maize by activating the antioxidant enzyme defense system and reducing the bioavailability of heavy metals [25]. Additionally, the application of MWCNTs has been shown to enhance biomass in various tissues of ramie (*Boehmeria nivea* L.) under cadmium (Cd) stress [26]. Simultaneously, in real-world environments, complex exposure scenarios arise under identical pollutant exposure conditions, yet studies on exposure sequences are still relatively scarce. Moreover, research concerning the combined phytotoxicity of MWCNTs and heavy metals is lacking. Therefore, a considerable gap remains in the current understanding of the environmental impacts of MWCNTs.

In this study, to investigate the toxic effects of three forms of MWCNTs (unmodified MWCNT, MWCNT-OH, and MWCNT-COOH) as well as Cu, we selected ryegrass as the model plant, which is a high-quality forage commonly utilized in animal husbandry and agricultural research [27]. We aimed to (1) investigate the impact of different types of MWCNTs on plant growth and physiology, (2) examine the effects of coexisting heavy metal ions and nanomaterials on plant health, particularly whether they induce synergistic or additive toxic effects, and (3) explore the influence of MWCNTs on the absorption and accumulation of Cu^2+^ in plants. We investigated the effects of MWCNTs with different functional groups on the biomass, chlorophyll content, and MDA levels of ryegrass. Also, under the combined exposure conditions with Cu, the effects of MWCNT concentration and exposure time pattern on ryegrass were explored over a period of 12 days. Furthermore, infrared spectroscopy was employed to elucidate the interaction among roots, MWCNTs, and Cu^2+^. This study provided evidence for assessing the ecological risks of MWCNTs combined with heavy metals and predicts the potential impacts of MWCNTs and Cu^2+^ on plants under different environmental conditions.

## 2. Materials and Methods

### 2.1. Chemical and Reagents

Hydroxyl-modified MWCNT (MWCNT-OH), carboxyl-modified MWCNT (MWCNT-COOH), and unmodified MWCNT (MWCNT) were all purchased from Shenzhen Nanoport Co., Ltd. (Shenzhen, China). The material exhibits a non-carbon impurity content of less than 3%, with a specific surface area ranging from 40 to 300 m^2^/g and a purity of 95 wt%. Transmission Electron Microscopy (TEM) was conducted to observe the morphology and structural characteristics of MWCNT and MWCNT-COOH (Figure S1a,b). All reagents used in this experiment were of analytical reagent (AR) grade purity.

### 2.2. Plant Culture and Treatments

Uniform and vigorous wide-leafed tetraploid monocarpic ryegrass seeds (*Lolium perenne Linn*.) were sterilized with 1.2% NaClO solution for 20 min and then thoroughly rinsed with deionized water. The seeds, covered with water-saturated filter papers, were cultured in Petri dishes for four days to germinate. Subsequently, the seedlings were thinned to one plant per flask in 1/2 Hoagland solution and grown in a controlled greenhouse with a day/night temperature of 27 ± 2/17 ± 2 °C, relative humidity of 50–70%, and light intensity of 50–80 µmol m^2^ s^−1^ for 16 h. After 21 days, the height, weight, and branch count were measured to ensure phenotypic similarity among selected individuals. The photograph of the exposure design is presented in Figure S2. A total of 192 ryegrass plants were utilized in the experiment.

In the individual exposure experiment, the concentrations of MWCNTs were set as 0, 20, 50, 100, 200, 500, and 1000 mg/L, and the concentrations of Cu^2+^ were set as 0, 5, 10, 20, 50, 100, and 200 mg/L. Meanwhile, to evaluate the effects of MWCNT functional groups on ryegrass, unmodified MWCNT, MWCNT-OH, and MWCNT-COOH were individually administered.

In the co-exposure experiment, three treatments were designed to investigate the influence of contaminant introduction timing on toxicity and interactions: (1) MWCNT added to Cu^2+^-containing culture medium after 24 h (Cu-24-MWCNT); (2) simultaneous introduction of MWCNTs and Cu^2+^ (Cu-MWCNT); (3) MWCNTs added 24 h before Cu^2+^ exposure (MWCNT-24-Cu). Cu concentration was set at 5 mg/L, and MWCNT concentrations were set at 0, 20, 50, 100, and 200 mg/L. Samples were collected at 3, 6, 9, and 12 days after exposure. Each treatment was performed in triplicate with three randomly selected ryegrass samples per group.

After 12 d of exposure, plant roots from each treatment were immersed in a 20 mmol EDTA solution to chelate and remove surface-bound Cu^2+^. Subsequently, they were rinsed sequentially to thoroughly remove any residual chemicals. Following the measurement of sample fresh weight, root systems were separated and root length was measured. After measuring the fresh weight and root length of the samples, the specimens were blanched at 110 °C for 30 min and dried at 80 °C for 24 h, and their dry weight was then recorded.

### 2.3. Determination of Chlorophyll Concentration

The finely ground ryegrass fresh samples were placed into test tubes with 10 mL extraction solution (equal volumes of acetone and anhydrous ethanol). After 24 h, when the sample had turned completely white, indicating complete extraction, the absorbance was measured at 663 nm and 645 nm using an ultraviolet-visible (UV-VIS) spectrophotometer. Chlorophyll content was calculated using the following formulas:

(1) $$C_{a}={12.70A}_{663}-{2.59A}_{645}$$

(2) $$C_{b}={22.88A}_{645}-{4.67A}_{663}$$

(3) $$C_{T}=C_{a}+C_{b}$$

where $C_{a}$, $C_{b}$, and $C_{T}$ are the concentration of chlorophyll A and chlorophyll B and the total chlorophyll in mg/g fresh weight.

### 2.4. Determination of MDA

A 0.6% TBA solution was prepared by dissolving 0.6 g of thiobarbituric acid in 100 mL of 10% TCA. Fresh ryegrass (0.2 g) was homogenized with 10 mL of 10% TCA and quartz sand, then centrifuged at 3000 rpm for 10 min. Then, 2 mL of the supernatant was mixed with an equal volume of 0.6% TBA, heated in a boiling water bath for 15 min, and then rapidly cooled. After a second centrifugation at 3000 rpm for 10 min, absorbance was measured at 450 nm, 500 nm, and 600 nm using a UV-VIS spectrophotometer. MDA concentration in the extraction solution was calculated using the following formula:

(4) $$C_{MDA1}=6.45\times \left(A_{532}-A_{600}\right)-0.56A_{450}$$

(5) $$C_{MDA2}=C\times V\times M^{-1}$$

where $C_{MDA1}$ is the volumetric molar concentration of MDA in the extraction solution, μmol/L. V is volume of the extraction solution, mL. M is the fresh weight or area of the leaves, g. $C_{MDA2}$ is the mass molar concentration of MDA in ryegrass, μmol/g.

### 2.5. Quantification of Cu in Ryegrass

Ryegrass samples were washed three times with deionized water, blanched at 110 °C for 30 min, and then dried at 80 °C to a constant weight. Dried ryegrass (0.1 g) was finely ground and placed into a digestion vessel with 7 mL of 68% HNO_3_ and 1 mL H_2_O_2_ for microwave digestion (ramped at 15 °C/min to 160 °C, then held for 30 min). The solution was then adjusted to 10 mL with 1% HNO_3_ and filtered through a 0.22 μm syringe filter for analysis. Cu concentration was determined using an atomic fluorescence spectrometry (AFS-9130, Beijing Titan Instruments Co., Ltd., Beijing, China) with highly intensive hollow cathode lamps (HCLs) for copper.

### 2.6. Data Analysis

Each treatment was replicated three times, and data are presented as mean ± standard deviation (SD). Statistical analysis was conducted using one-way ANOVA to assess differences among the treatment groups. Tukey’s post hoc test was applied for multiple comparisons with a significance threshold of *p* < 0.05. All analyses were performed using Statistical Product and Service Solutions 29.0 (SPSS 29.0) software.

## 3. Results and Discussion

### 3.1. Effect of Individual MWCNT on the Biomass, Chlorophyll Content, and MDA Level of Ryegrass

**Figure 1:**
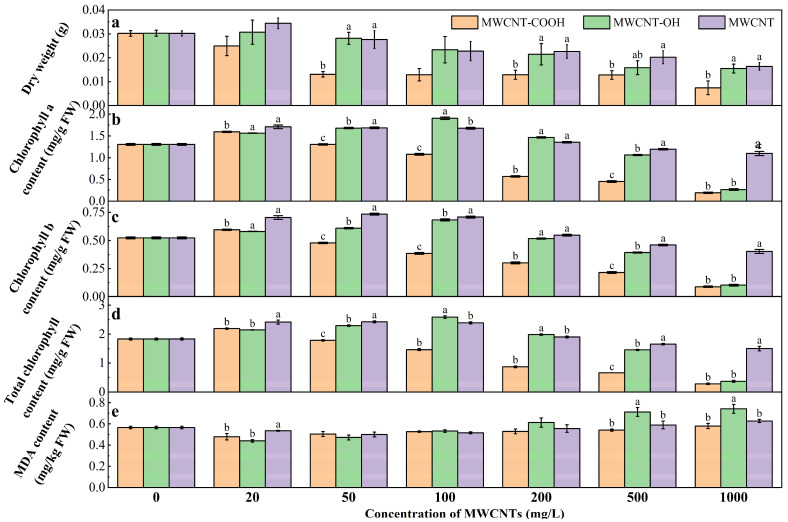
Effects of MWCNT-COOH, MWCNT-OH, and unmodified MWCNT on the dry weight (**a**), chlorophyll a content (**b**), chlorophyll b content (**c**) total chlorophyll content (**d**), and MDA content (**e**) of ryegrass (Different letters denote significant differences (*p* < 0.05) across treatments at the same concentration, as revealed using one-way analysis of variance (ANOVA) followed by the Tukey’s post hoc test, the same as follows).

TEM images show that MWCNTs had inner and outer diameters of approximately 10 ± 0.5 nm and 20 ± 1 nm, respectively, with lengths ranging from 1 to 2 μm (Figure S1). The impact of three MWCNTs on the biomass of ryegrass is illustrated in Figure 1a. Under 20 mg/L MWCNT exposure, an increase in biomass was observed, indicating that low doses of MWCNTs might promote ryegrass growth. Under 1000 mg/L MWCNT exposure, the biomass of ryegrass was reduced by 41% compared to the control. MWCNTs exhibit significant toxicity to cells even at microgram levels [28,29]. Generally, a noticeable inhibition of ryegrass biomass was observed with elevations in concentration. The inhibitory effect was also confirmed by observing yellowing of stems and leaves, necrosis of root tips, wilting, and substantial shedding of the root system at high concentrations. MWCNTs suppressed the growth of various plant species. For example, negatively charged SWCNTs, which are functionalized with poly-3-aminobenzenesulfonic acid, have been found to hinder root elongation in lettuce and tomatoes [30].

The contents of chlorophyll a (Chl a) and chlorophyll b (Chl b) in ryegrass are shown in Figure 1b,c. Increasing contents of MWCNTs generally decreased the Chl a and Chl b contents in leaves. The Chl a and Chl b contents were decreased by 85.19% and 94.43% under 1000 mg/L MWCNT-COOH compared to the control without MWCNTs. Notably, MWCNT-COOH further reduced the Chl a and Chl b concentrations in ryegrass compared to the unmodified MNCNT and MWCNT-OH. Specifically, 1000 mg/L MWCNT-COOH decreased the Chl a and Chl b contents by 82.37–78.13% and 27.48–14.3% compared to unmodified MWCNT and MWCNT-OH, respectively. Thus, MNCNTs, especially MNCNT-COOH, impaired the photosynthetic capacity of ryegrass. Similarly, exposure to ZnO nanoparticles for 6 days reduced 33% of the Chl a content and 71.8% of the Chl b content in *Azolla filiculoides* [31]. The reduction in Chl a content caused by MNCNTs can affect the electron transport efficiency in the light reactions, particularly inhibiting photosystem II, since chlorophyll a is directly involved in the reactions of both photosystem I and II. Meanwhile, the decrease in Chl b content under MNCNTs exposure can lead to a diminished capacity to capture a broader spectrum of light [32,33].

Figure 1d illustrates the impact of MWCNTs on the total chlorophyll content of ryegrass. As the concentrations of MWCNT and MWCNT-COOH increased from 20 mg/L to 100 mg/L, the total chlorophyll content in plants increased, which was consistent with the Chl a and Chl b. However, MWCNT-COOH caused a dose-dependent decrease in chlorophyll content in ryegrass under concentrations of MWCNTs over 1000 mg/L, which was similar to the effect of MWCNTs on *Arabidopsis* T87 suspension cells. The reduction in plant chlorophyll content impacts photosynthesis, thereby hindering or impairing plant growth [34]. The carboxyl groups in MWCNTs enhance interaction with cellular components through stable chemical bonds [35], potentially causing cell membrane disruption and oxidative stress in plant cells.

Furthermore, the physicochemical properties of the material itself also play a decisive role. MWCNTCOOH caused the greatest inhibition to ryegrass biomass and chlorophyll content, while unmodified MWCNTs had the least negative impact (Figure 1a,b). Compared to the control, the biomass of the ryegrass treated with 1000 mg/L of MWCNT-COOH, MWCNT-OH, and unmodified MWCNT decreased by 75.33%, 48.43%, and 45.4%, respectively. Meanwhile, in comparison with the unmodified MWCNT group, the chlorophyll contents of the MWCNT-COOH and MWCNT-OH groups declined by 61.02% and 75.38%, respectively.

The modification type significantly impacted MWCNT biocompatibility and toxicity. COOH group acidity tended to disturb plant cell pH homeostasis, exacerbating internal stress and posing higher risks due to increased bioactivity. OH-modified MWCNTs, less reactive due to weaker -OH group interactions, show lower toxicity. Unmodified MWCNTs, due to their more inert surface [36], primarily impact plants through physical actions such as penetration or blockage rather than chemical reactions.

With the increasing concentration of MWCNT-OH and unmodified-MWCNT from 0 to 1000 mg/L, the MDA contents of ryegrass were gradually increased by 32.39% and 11.96% (Figure 1e), indicating oxidative damage to the plant tissues caused by MWCNTs. Notably, exposure to different concentrations of MWCNT-COOH alone had no effect on the MDA content in ryegrass. The cell toxicity of MWCNT is closely related to its physicochemical properties, such as surface modification and dispersion in the solution. The surface properties of nanoparticles are a key factor in determining the interaction between nanoparticles and cells [37]. MWCNT-OH is more dispersed and water-soluble, and as such it could reach the nucleus by crossing undamaged membranes and interact with DNA [38], thereby more easily causing oxidative stress damage.

### 3.2. Effect of Individual Cu^2+^ on the Biomass, Chlorophyll Content, and MDA Level of Ryegrass

Compared to the control, the toxicity of Cu^2+^ to ryegrass resulted in a gradual reduction in biomass with increasing Cu^2+^ concentrations (Figure S3a). Specifically, compared to the control, at Cu^2+^ concentrations of 100 and 200 mg/L, the biomass decreased by 85.7% and 91.4%, respectively. Heavy metals disrupt plant metabolism, inhibit growth, and interact with polysaccharides in the plant cell wall, reducing elasticity and making roots more sensitive to metal pollution [39,40]. Moreover, exposure to Cu^2+^ significantly decreased the chlorophyll content in ryegrass with increasing concentrations of Cu^2+^ (Figure S3b). In comparison to the control, Cu^2+^ (from 10 to 200 mg/L) distinctly reduced the chlorophyll content. High concentration of Cu^2+^ can inhibit chlorophyll synthesis and disrupt chloroplast structure [41,42]. As shown in Figure S3c,d, both Chl a and Chl b contents were decreased with the increasing Cu concentrations, indicating that Cu could inhibit the light energy utilization efficiency of ryegrass and electron transport [43]. Similarly, the reduction in total Chl, Chl a, and Chl b contents of mustard (*Brassica juncea*) treated with increasing concentrations of Cd (0–100 mg/kg) also exhibited a dose-dependent relationship [44].

In addition, the MDA contents in ryegrass were significantly enhanced with increasing concentrations of Cu^2+^ (Figure S3e), with the 200 mg/L Cu^2+^ group showing a 38.98% increase compared to the control. The malondialdehyde (MDA) indicator is capable of reflecting the degree of membrane lipid peroxidation damage caused by ROS [45,46]. The bioaccumulation of MDA suggested a high oxidative state of ryegrass after it was exposed to MWCNTs. Figure S3f shows that there is a positive correlation between Cu^2+^ concentration and Cu content in ryegrass roots. In addition, Cu stress significantly elevated the production rate of O_2_^−^, increased H_2_O_2_ levels, and enhanced MDA accumulation in the leaves of alfalfa (*Medicago sativa*) [47]. However, in spinach seedlings, MDA content showed no significant difference (*p* > 0.05) when the plants were treated with CuSO_4_ at a concentration of 100 mg/L, suggesting variation in MDA’s effect across different species [48].

### 3.3. Synergistic Impact of MWCNT and Cu^2+^ on the Biomass of Ryegrass

**Figure 2:**
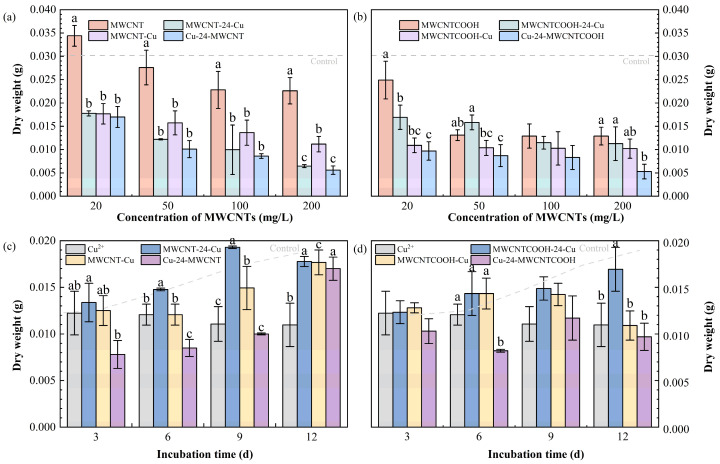
The combined effect of different MWCNT concentrations on dry weight, showing the relationship between biomass and cultivation time exposure time patterns: (**a**) unmodified MWCNT; (**b**) MWCNTCOOH. Comparison of the effects of MWCNTs addition time pattern on the dry weight of ryegrass with the exposure days: (**c**) unmodified MWCNT; (**d**) MWCNTCOOH. The dashed line represents the value of the control group (treated without MWCNTs and Cu^2+^). Different letters denote significant differences (*p* < 0.05) across treatments at the same concentration, as revealed using one-way analysis of variance (ANOVA) followed by the Tukey’s post hoc test.

Figure 2a,b depict the comparative impact of varying concentrations of MWCNT (or MWCNT-COOH) in conjunction with Cu^2+^ on ryegrass biomass. Meanwhile, the combined effect of MWCNTs and Cu^2+^ on the biomass of ryegrass over 12 days is shown in Figure 2c,d. At a concentration of 20 mg/L, the dry weight of ryegrass exposed to the combined treatment of unmodified MWCNTs decreased by 48.5% compared to the single exposure (Figure 2a). At an equivalent concentration, the reduction in dry weight for ryegrass subjected to complex treatments with MWCNT-COOH was observed to range from 32% to 61.09% compared to the group treated solely with MWCNT-COOH (Figure 2b). Particularly, at a higher concentration of 200 mg/L of unmodified MWCNTs, the dry weight of ryegrass treated with MWCNT-24-Cu, MWCNT-Cu, and Cu-24-MWCNT dramatically declined by 71.37%, 48.23%, and 75.22%, respectively, compared to the MWCNT group (Figure 2a). Therefore, it can be concluded that the co-exposure to MWCNTs and Cu drastically decreased the ryegrass biomass compared to the individual MWCNT exposure.

Moreover, the pre-exposure to MWCNTs (50–200 mg/L) followed by Cu^2+^ caused a less severe impact on biomass production than Cu single exposure. On the 12th day, the biomass of the MWCNT-Cu and MWCNTCOOH-Cu treatments showed individual increases of 38.42% and 35.5% over the individual Cu group. The reduction in Cu^2+^ concentration in the ryegrass, likely due to Cu^2+^ adsorption by MWCNTs in the culture medium, may have alleviated the toxic effect on the plant. Meanwhile, CNTs have been reported to mitigate the impact of copper stress on the germination and growth of corn seeds. Co-exposure to Cu^2+^ and CNTs or carbon nanoparticles (CNPs) under 100 mg/L significantly reduced Cu stress, especially at higher concentrations of Cu^2+^ [49].

Under the combined exposure circumstances, the biomass of ryegrass decreased significantly by 38.9–58.8% when the concentration of MWCNTs rose from 20 to 1000 mg/L (Figure 2a,b). This result is consistent with the combined effect of MWCNTs and Cd on wheat [6]. In general, under co-exposure, MWCNT-COOH had a more significant negative impact on the biomass of ryegrass than unmodified MWCNTs. Specifically, as the concentration of MWCNT increased (20, 50, 100, 200 mg/L), the biomass of ryegrass treated with unmodified MWCNT was 62.29%, 48.31%, 32.72%, and 9.51% higher than that treated with MWCNT-COOH, respectively (Figure 2c,d).

Over the 12-day cultivation, ryegrass biomass in most co-exposure groups improved incrementally yet consistently lagged behind the control (Figure 2c,d). MWCNT-24-Cu and MWCNTCOOH-24-Cu demonstrated an increase of 47.5% and 41.08%, respectively. However, the Cu-24-MWCNTCOOH group experienced a significant biomass reduction of 19.17%, suggesting that pre-exposure to Cu^2+^ may cause higher toxicity to plants than pre-exposure to MWCNT. This reduction might be attributed to the plant’s hormonal response, specifically the release of abscisic acid (ABA), which induces the senescence of affected leaves and roots as a protective mechanism against the detrimental effects of environmental pollutants. By reducing metabolic activity and nutrient transport in the affected tissues, the plant helps to concentrate its resources and energy to cope with harmful environmental conditions [50].

### 3.4. Synergistic Impact of MWCNT and Cu^2+^ on the Chlorophyll Content of Ryegrass

**Figure 3:**
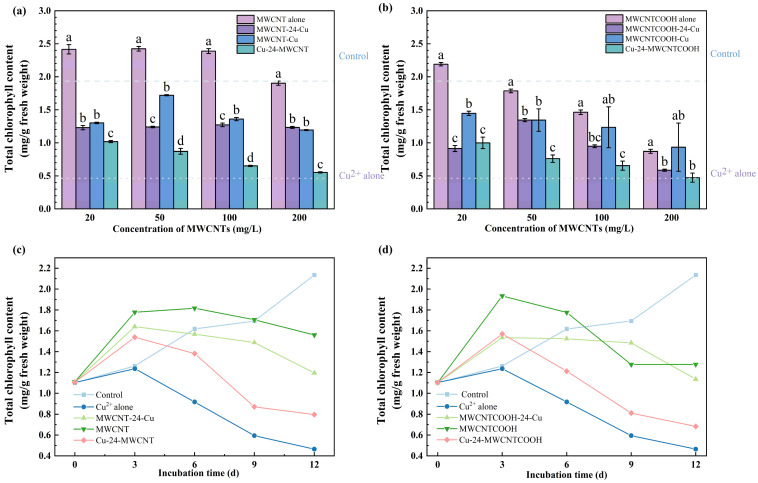
Impact of MWCNT concentration under combined effects on total chlorophyll content: (**a**) unmodified MWCNT, (**b**) MWCNTCOOH. Comparison of the effects of MWCNTs addition time pattern on the total chlorophyll content with the exposure days: (**c**) unmodified MWCNT, (**d**) MWCNTCOOH. The dotted line represents the value of the control group (treated with only Cu^2+^ and without MWCNT). The dashed line represents the value of the Cu^2+^-alone group (treated without MWCNT and Cu^2+^). Different letters denote significant differences (*p* < 0.05) across treatments at the same concentration, as revealed using one-way analysis of variance (ANOVA) followed by the Duncan’s post hoc test.

After 12 days of toxic treatment, the effect of the combined action of MWCNT at different concentrations on ryegrass chlorophyll is illustrated in Figure 3a,b. Compared to single exposure to Cu, the introduction of unmodified MWCNT and MWCNT-COOH in concentrations ranging from 20 to 200 mg/L resulted in an increase in chlorophyll content by 20.11% to 272.98% and 42.67% to 213.67%, respectively. However, in contrast to the single exposure system of MWCNT, the combined exposure enhanced the toxic effect on chlorophyll. The addition of 20 mg/L of unmodified MWCNT and MWCNT-COOH to the system resulted in an average reduction of 51.07% and 48.9%. Combined pollution exacerbated the impact of single MWCNT on the important indicator of plant photosynthesis, chlorophyll, but mitigated the harm of single Cu, aligning with the trend observed in biomass reduction. Coincidentally, in a study examining the effects of MWCNT and copper on the microalga (*Skeletonema costatum*) [51], it was found that the toxicity of CNTs on the microalgae was significantly lower than that of nano Cu.

Based on the Pearson correlation analysis presented in Table S1, there was a negative correlation between the concentration of MWCNTs in the system and the chlorophyll content of ryegrass. This correlation was particularly pronounced in the scenario where Cu^2+^ was introduced first. After the concentration of unmodified MWCNT and MWCNT-COOH increased from 20 to 200 mg/L, the chlorophyll content decreased by 45.58% and 52.58%, respectively. This also reflects that MWCNT-COOH has a more detrimental impact on plant physiology. The dose–effect data of MWCNT-Cu and MWCNTCOOH-24-Cu indicate that, under low concentrations of MWCNTs, plants exhibit a stimulatory response, leading to an increase in chlorophyll synthesis. This phenomenon aligns with the earlier findings in plants [52,53]. In adverse stress conditions, ryegrass activates its internal stress response systems, accelerating metabolism to eliminate internal stressors. This stimulation results in an initial increase in chlorophyll synthesis. The impact of MWCNT concentration under co-pollution with Cu on Chl a and Chl b contents is presented in Figure S4. In alignment with the results discussed in Section 3.1, both Chl a and Chl b are negatively impacted. Under 200 mg/L MWCNT exposure, the levels of Chl a and Chl b in Cu-24-MWCNT and Cu-24-MWCNTCOOH decreased by 70.09% and 72.93% and by 39.22% and 57.85%, respectively, compared to single MWCNT and MWCNTCOOH exposure.

Compared to other introduction timing treatments, the priority introduction of Cu^2+^ significantly exacerbated the inhibitory effect on the chlorophyll content of ryegrass. At an MWCNT concentration of 100 mg/L, adding Cu first reduced the chlorophyll content by 48.08% compared to adding unmodified MWCNT first and by 51.48% compared to adding Cu and unmodified MWCNT simultaneously. For MWCNT-COOH, the reduction in chlorophyll content was slightly more moderate when Cu was added first, decreasing by 30.91% compared to the system where MWCNT-COOH was added first and by 47.91% when added simultaneously.

Figure 3c,d show that under the combined influence of MWCNT and Cu^2+^, chlorophyll content in ryegrass gradually decreased within 12 days but remained higher than Cu^2+^ alone group. On the third day, the chlorophyll contents of the six compound-polluted groups significantly increased, even surpassing the control group by 21.63% to 53.49%. This is likely due to the initial resistance of ryegrass to stress during the early stages. However, the chlorophyll content then gradually showed a downward trend, with the Cu-24-MWCNT groups exhibiting the most pronounced decline.

Research has indicated that when heavy metal ions such as Cu^2+^ enter plant tissues, they interact with proteins by binding to or substituting for Mg^2+^ and Fe^2+^ in chloroplasts [54]. Cu^2+^ enhances peroxidase, which decomposes hydrogen peroxide and contributes to chlorophyll degradation, thereby directly or indirectly accelerating the breakdown of chlorophyll. This interaction affects key enzymes in the chlorophyll biosynthetic pathway, such as chlorophyll reductase and protochlorophyllide deaminase, altering their structures and inhibiting their activities, thereby impeding chlorophyll synthesis. Additionally, higher concentrations of MWCNT may reduce Na^+^ and K^+^-ATPase activity to inhibit chlorophyll synthesis in ryegrass [55].

### 3.5. Effect of MWCNT and Cu^2+^ on the MDA Level of Ryegrass

**Figure 4:**
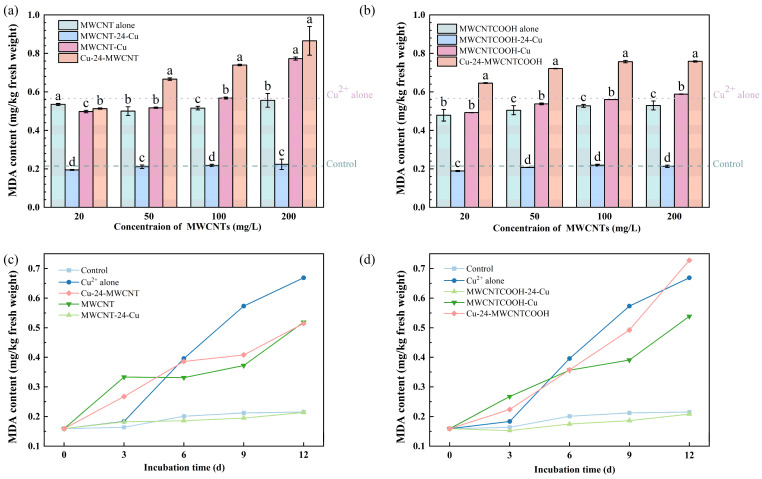
Impact of MWCNT concentration under combined effects on MDA content: (**a**) unmodified MWCNT, (**b**) MWCNTCOOH. Comparison of the effects of MWCNTs addition time pattern on the MDA content with the exposure days: (**c**) unmodified MWCNT, (**d**) MWCNTCOOH. The dashed line represents the value of the control group, which was treated without MWCNT and Cu^2+^. The dotted line represents the value of the group, which was treated with only Cu^2+^ and without MWCNT (Different letters denote significant differences (*p* < 0.05) across treatments at the same concentration, as revealed using one-way analysis of variance (ANOVA) followed by the Tukey’s post hoc test).

Figure 4a,b illustrate that after a 12-day co-exposure to MWCNT and Cu, MDA levels in ryegrass escalate with increased MWCNT concentrations. Notably, at the concentration of 50 to 1000 mg/L, the MDA of MWCNT-Cu, MWCNTCOOH-Cu, Cu-24-MWCNT, and Cu-24-MWCNTCOOH increased by 19.49%, 12.69%, 69.46%, and 17.46%.

However, the MDA levels in the MWCNTs priority exposure group always remained close to those of the control group and were even significantly lower than those in the MWCNT single exposure group. The observed phenomenon may be attributed to two factors. First, MWCNTs adsorb Cu^2+^, reducing its availability for and absorption by ryegrass roots, thus mitigating Cu^2+^ toxicity. Second, ryegrass roots secrete amino acids in response to stress, which can chelate Cu^2+^ and further decrease its concentration in the culture medium [56].

After 12 days of combined exposure to MWCNT and Cu in ryegrass, Figure 4c,d reveal a general trend of increased MDA content over time. During the cultivation period, both the control group and the MWCNTs priority exposure group showed a low and stable trend in MDA content, while other compound exposure groups exhibited a rapid upward trend in MDA content of ryegrass roots. For instance, when Cu was added simultaneously with unmodified MWCNTs (as well as MWCNT-COOH), the MDA content increased by 3.23 and 4.57 times within 12 days, respectively. When plant organs undergo senescence or sustain damage under adverse conditions, lipid peroxidation often occurs, and MDA is the ultimate decomposition product of lipid peroxidation. The content of MDA serves as an important indicator of the extent of damage induced by adverse environmental conditions on plants [57,58].

### 3.6. Effect of Co-Exposure of MWCNT and Cu^2+^ on Cu Absorption and Bioaccumulation in Ryegrass

**Figure 5:**
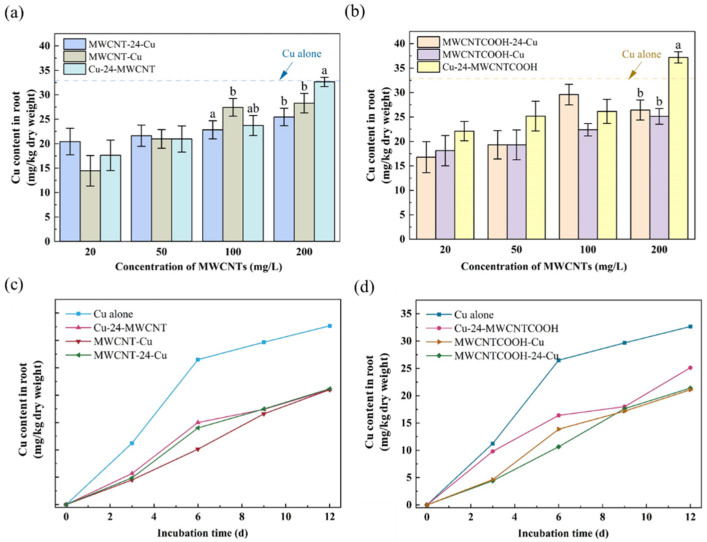
Comparison of the effects of MWCNT concentration on the Cu^2+^ adsorption on root: (**a**) unmodified MWCNT; (**b**) MWCNTCOOH. Comparison of the effects of MWCNTs addition time pattern on the ryegrass root uptake of Cu^2+^ with the exposure days: (**c**) unmodified MWCNT; (**d**) MWCNTCOOH. The dashed line represents the value of the group treated with only Cu^2+^ and without MWCNT (Different letters denote the significant difference (*p* < 0.05) across treatments at the same concentration, as revealed using one-way analysis of variance (ANOVA) followed by the Tukey’s post hoc test).

Under the treatment of combined MWCNT and Cu^2+^, as shown in Figure 5a,b, Cu^2+^ in the root rises sharply with the increase in MWCNT concentration but is generally lower than the concentration in the Cu alone group. It is noteworthy that at a concentration of 200 mg/L of MWCNT-COOH, the root Cu content in the Cu-24-MWCNTCOOH group was 13.12% higher compared to the group exposed to Cu alone. As the concentration of MWCNTs increased from 20 mg/L to 200 mg/L, the Cu content in the roots of ryegrass in the MWCNT-24-Cu, MWCNT-Cu, and Cu-24-MWCNT groups increased by 24.64%, 95.65%, and 85.27%, respectively. Concurrently, within the MWCNT-COOH-exposed groups, the levels of Cu increased on a comparative basis (relative to the group treated with Cu^2+^ individually) by 57.35%, 38.60%, and 68.32% across the three different time patterns. This may be due to the fact that MWCNTs, as solid particulate pollutants, are more likely to adhere to the surface of the ryegrass roots after adsorbing Cu^2+^. This adhesion may provide more favorable conditions for the migration of Cu^2+^ into the ryegrass roots. Consequently, MWCNTs can further alter the balance between plants and toxic metals in the environment, thereby promoting the transport of heavy metals (loids) into the plant system [59].

In general, the preferential exposure to Cu^2+^ facilitated the absorption of Cu in the roots of ryegrass. At 200 mg/L MWCNTs, in the unmodified MWCNT group, the Cu content in the group with Cu-prioritized exposure was 40.8% and 48.02% higher than in the simultaneous exposure and Cu-delayed exposure groups; for the MWCNT-COOH groups, these differences were 28.23% and 15.46%, respectively. It is precisely because of the preferential exposure to Cu^2+^ that an increased absorption of heavy metals may have occurred, which in turn affected the biomass, chlorophyll content, and degree of damage in ryegrass.

Figure 5c,d illustrate the relationship between the Cu content in the roots and the cultivation time under various exposure time patterns. During the experimental period from 0 to 12 days, it was observed that under both single and complex exposure conditions, the copper (Cu) content in the organisms showed a significant linear increase with the advancement of time. On the 12th day, the root Cu content in the groups exposed to Cu-prioritized exposure, Cu-coincidental exposure, and Cu-delayed exposure with unmodified MWCNT (and MWCNT-COOH) was reduced by 46.42%, 55.51%, and 53.84% (23.11%, 54.76%, and 53.73%, respectively) compared to the single Cu exposure group. Thus, the introduction of MWCNTs significantly reduced the uptake of Cu in the roots. This phenomenon not only mitigates the potential toxic effects of heavy metals on plants but also provides an effective strategy for regulating plant absorption of heavy metals.

Additionally, under conditions of complex exposure, the groups treated with MWCNT-COOH exhibited a significant increase in root Cu content compared to those treated with unmodified MWCNT. On the 12th day, the content in the Cu-prioritized exposure group increased by 19.57% compared to the unmodified MWCNT (Figure 5c,d). The presence of carboxyl groups (-COOH) on the surface of MWCNT-COOH enhances its binding affinity to Cu ions, thereby facilitating the adsorption and bioaccumulation of Cu in the roots. Additionally, the introduction of these hydrophilic carboxyl groups may improve the dispersibility of MWCNT-COOH in the root environment and strengthen its interaction with the plant roots.

### 3.7. Interaction Between MWCNTs and Cu^2+^ in Nutrient Solution

**Figure 6:**
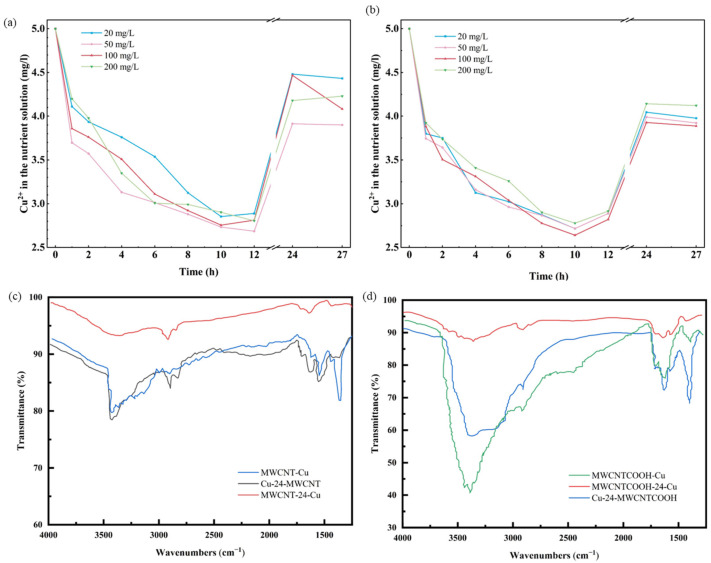
The impact of MWCNTs at varying concentrations on the level of Cu^2+^ in a copper-enriched nutrient solution: (**a**) unmodified MWCNT; (**b**) MWCNT-COOH. Infrared spectra comparison of different MWCNTs introduced at different time points: (**c**) unmodified MWCNT; (**d**) MWCNT-COOH.

Figure 6a,b illustrate the distinct trend of Cu^2+^ adsorption by MWCNTs in the nutrient solution. At first (0–8 h), the concentration of Cu^2+^ decreases due to rapid adsorption by MWCNTs, then increases as Cu^2+^ is released from MWCNTs (8–12 h), eventually representing an equilibrium state (12–27 h). While the Cu^2+^ adsorption trends in 27 h align between unmodified MWCNT and MWCNT-COOH, at equilibrium, the Cu^2+^ concentration in the unmodified MWCNT culture medium surpasses that in the MWCNT-COOH culture medium. This implies that unmodified MWCNT exhibits a superior adsorption capacity for Cu^2+^ compared to MWCNT-COOH. This may be attributed to unmodified MWCNT lacking functional groups, providing more unoccupied active sites and allowing Cu^2+^ greater exposure to the MWCNT surface for adsorption.

The infrared spectroscopy of the hydroponic nutrient solution of ryegrass was shown in Figure 6c,d. The peak at 3410 cm^−1^ corresponds to -OH, while the peaks at 1720 cm^−1^ and 1640 cm^−1^ correspond to >C=O [60], representing subordinate and main carbonyl groups, respectively. The -CO-N double peaks are observed at 3350 cm^−1^ and 3200 cm^−1^, while the -N-O peak appears at 1620 cm^−1^. According to the functional group analysis, this substance is likely to be an amino acid. Therefore, we hypothesize that ryegrass may secrete amino acids from its roots to resist the combined pollution of MWCNTs and Cu.

Traditional perspectives suggest that root exudates can facilitate pollutant removal through two mechanisms: (1) enzymatic degradation of pollutants and (2) enhancement of microbial populations and activities to accelerate pollutant degradation [61]. However, studies have indicated another pathway through which ryegrass roots subjected to sole Cu^2+^ exposure may release amino acids, such as proline, glutamic acid, lysine, and phenylalanine. Conversely, the roots of ryegrass under sole MWCNT exposure may secrete organic acids or free radicals. Previous research has confirmed the presence of organic acids, with oxalic acid being the predominant low-molecular-weight organic acid, constituting over 98% of the total acids [62]. Consequently, the combined action of MWCNT and Cu^2+^ may induce ryegrass roots to secrete small-molecule organic acids and amino acids as a response to stress. With the increase in MWCNT concentration, the secretion of organic substances also increases. These organic acids form complexes with Cu^2+^ and remain in the solution, preventing the migration of Cu^2+^ from the environment into plants, ultimately mitigating toxicity.

## 4. Conclusions

The present study elucidates the complex toxicological interactions between MWCNTs and Cu^2+^ when co-exposed to ryegrass, revealing significant implications for environmental health and agricultural sustainability. Our findings indicate that MWCNTs, particularly at higher concentrations, can induce oxidative stress and growth inhibition in ryegrass, which are further exacerbated by the coexistence of Cu^2+^. However, at lower concentrations, MWCNTs can ameliorate the toxic effects of Cu^2+^, suggesting a protective role under certain conditions. Moreover, the combined exposure revealed a complex interaction: low concentrations of MWCNTs mitigated Cu^2+^ toxicity, whereas higher concentrations intensified it. As incubation continued, the Cu in the roots showed ongoing bioaccumulation. The impact of MWCNT surface functionalization is also highlighted, with MWCNT-COOH showing a more pronounced negative effect due to its negative charge properties. Infrared analysis indicated that ryegrass roots secreted organic or amino acids as a defense mechanism against these external stressors. This study emphasizes the role of nanomaterial–HM interactions in agricultural environments and the need for strategies to manage their ecological impact to protect ecological health. Future research should focus on developing mitigation strategies to minimize the ecotoxicological risks associated with nanomaterial and heavy metal co-exposure, ensuring ecological safety and agricultural productivity.

## Data Availability

The data supporting the findings of this study are available upon request.

## Supplementary Materials

The following supporting information can be downloaded at: https://www.mdpi.com/article/10.3390/nano14211746/s1, Figure S1. TEM images of (a) MWCNT; (b) MWCNT-COOH. Figure S2. Exposure design of the study. Figure S3. Comparison of the effects of Cu^2+^ on ryegrass. Figure S4. Impact of MWCNT concentration under combined effects on chlorophyll a content (a,c) and chlorophyll b content (b,d) of unmodified MWCNT (a,b), and MWCNT-COOH (c,d). Table S1. Pearson Correlation Analysis of MWCNT Concentration and Chlorophyll Content in Ryegrass.
