# Supplementary material for: Combined Toxicity of Multi-Walled Carbon Nanotubes and Cu2+ on the Growth of Ryegrass: Effect of Surface Modification, Dose, and Exposure Time Pattern
Source: Nanomaterials (Basel). 2024 Oct 30;14(21):1746. doi: 10.3390/nano14211746 (PMC11547606; doi:10.3390/nano14211746)
Supplement: Supplementary file 1 [file nanomaterials-14-01746-s001.zip › nanomaterials-3203480-supplementary.pdf]

Combined Toxicity of Multi-Walled Carbon Nanotubes and Cu^{2+} on the Growth of Ryegrass: Effect of Surface Modification, Dose, and Exposure Sequence

Wenwen Xie1, Cheng Peng1,2*, Weiping Wang1, Xiaoyi Chen1, Jiaqi Tan3*, and Wei Zhang1

1 *State Environmental Protection Key Laboratory of Environmental Risk Assessment and Control on Chemical Process, School of Resource and Environmental Engineering, East China University of Science and Technology, Shanghai 200237, China*
2 *Shanghai Institute of Pollution Control and Ecological Security, Shanghai 200092, China*
3 *Department of Biological Sciences, Louisiana State University, Baton Rouge, LA 70803, USA*
* Correspondence: (cpeng@ecust.edu.cn); Jiaqi Tan (jtan7@lsu.edu).

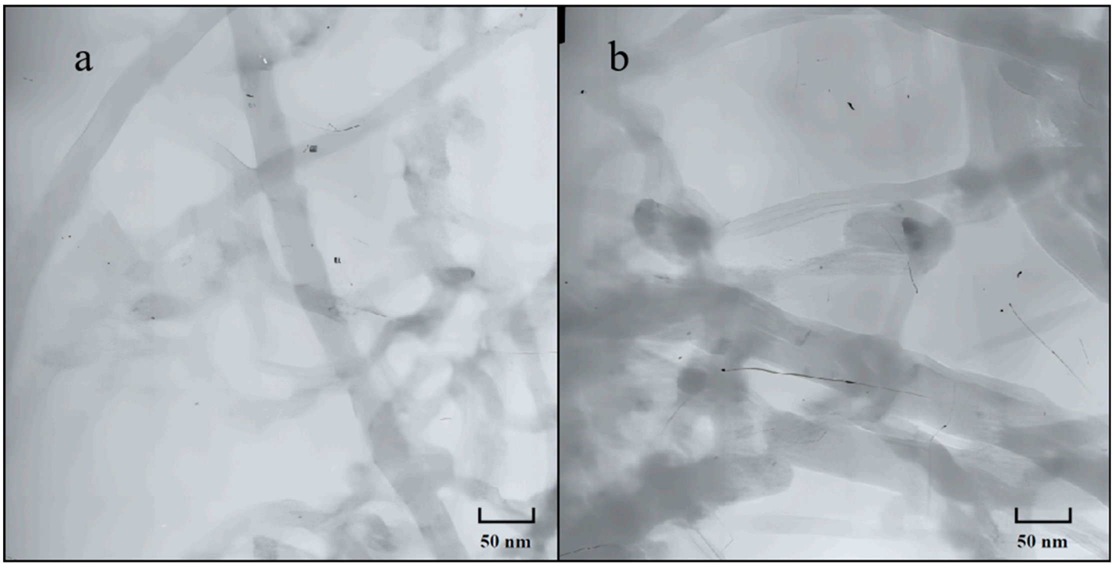

Figure S1. TEM images of (a) MWCNT; (b) MWCNT-COOH.

Citation: To be added by editorial staff during production.

Academic Editor: Fernanda Cássio

Received: 27 August 2024
Revised: 27 October 2024
Accepted: 28 October 2024
Published: 30 October 2024

Copyright: © 2024 by the authors. Submitted for possible open access publication under the terms and conditions of the Creative Commons Attribution (CC BY) license (https://creativecommons.org/licenses/by/4.0/).

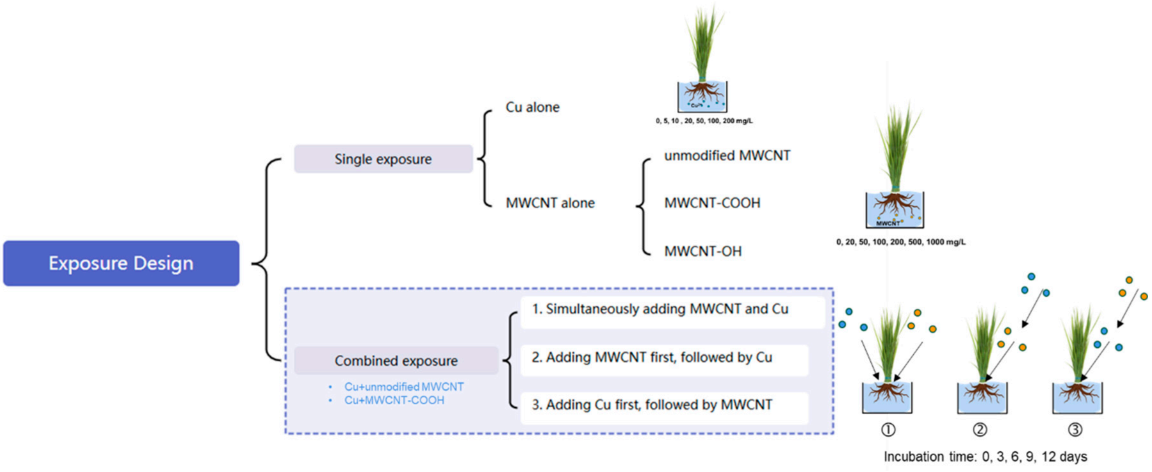

Figure S2. Exposure design of the study

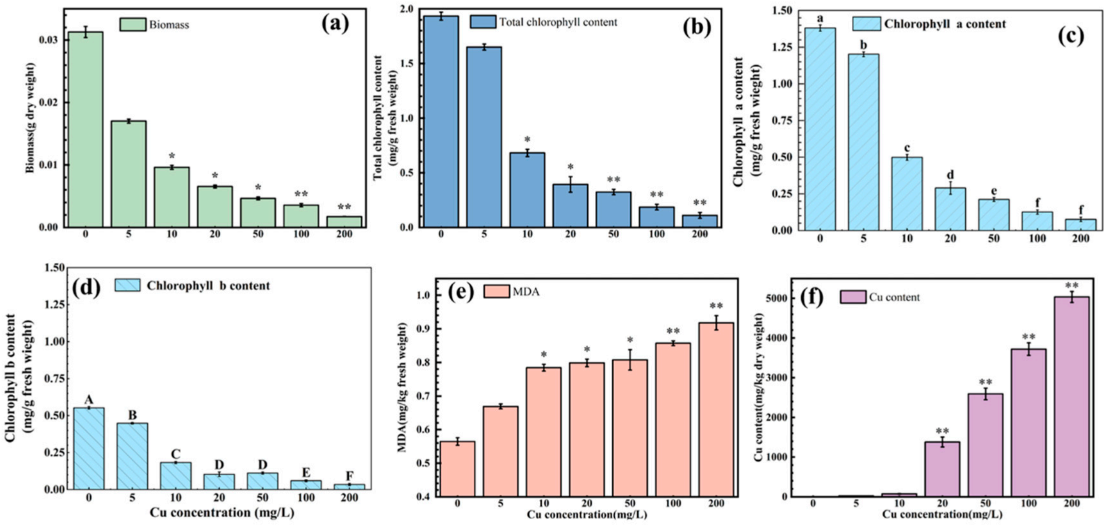

Figure S3. Comparison of the effects of Cu^{2+} on ryegrass: biomass (a), total chlorophyll content (b), chlorophyll a content (c), chlorophyll b content (d), MDA content (e) of the ryegrass and the Cu^{2+} concentration in the root of the ryegrass (f). (The asterisk * indicates a significance level of $p< 0.05$ and ** indicates $p< 0.01$, as revealed using one-way analysis of variance (ANOVA) followed by the Turkey's post-hoc test.)

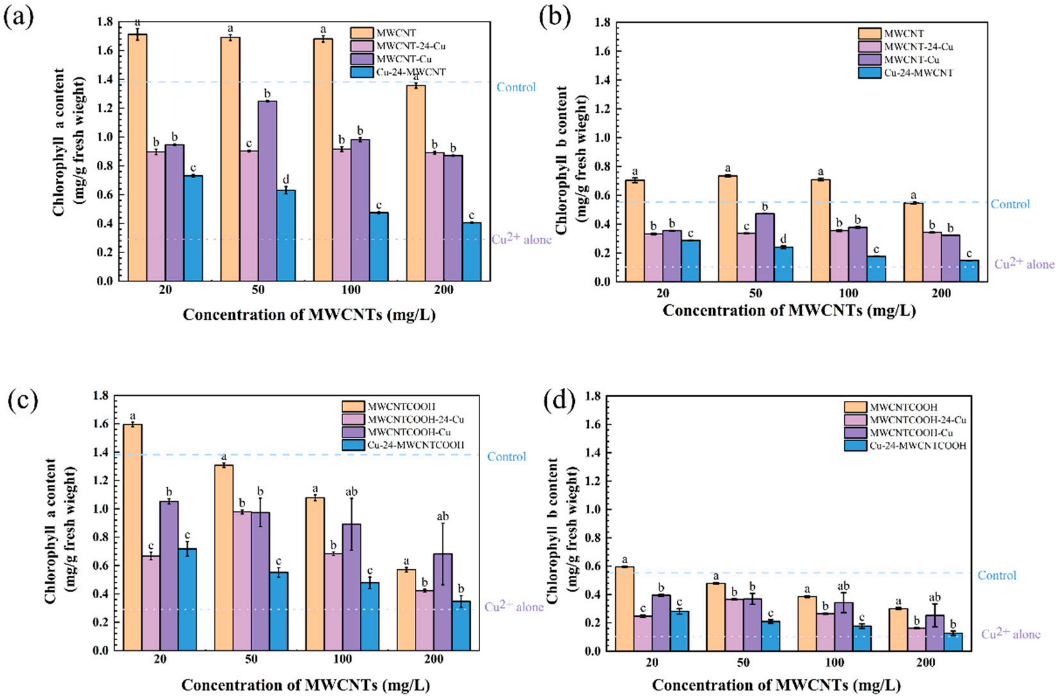

Figure S4. Impact of MWCNT concentration under combined effects on chlorophyll a content (a and c) and chlorophyll b content (b and d) of unmodified MWCNT (a and b), and MWCNT-COOH (c and d). The dot line represents the value of the control group (treated with only Cu^{2+} and without MWCNT). The dashed line represents the value of the Cu^{2+}-alone group (treated without MWCNT and Cu^{2+}). Different letters denote the significant difference ($p < 0.05$) across treatments at the same concentration, as revealed using one-way analysis of variance (ANOVA) followed by the Duncan's post-hoc test.

Table S1. Pearson Correlation Analysis of MWCNT Concentration and Chlorophyll Content in Ryegrass

Treatments	MWCNTs concentration	
	Pearson Correlation	-0.94*
MWCNTCOOH-24-Cu	Sig. (2-tailed)	0.017
	N	5
	Pearson Correlation	-0.234
MWCNTCOOH-Cu	Sig. (2-tailed)	0.704
	N	5
	Pearson Correlation	-0.777
Cu-24-MWCNTCOOH	Sig. (2-tailed)	0.122
	N	5
MWCNT-24-Cu	Pearson Correlation	-0.920*

	Sig. (2-tailed)	0.027
	N	5
	Pearson Correlation	-0.772
MWCNT-Cu	Sig. (2-tailed)	0.126
	N	5
	Pearson Correlation	-0.77
Cu-24-MWCNT	Sig. (2-tailed)	0.128
	N	5

The symbol * indicates a significant correlation at the 0.05 level (two-tailed).
